# Absolute Humidity and the Seasonal Onset of Influenza in the Continental US

**DOI:** 10.1371/currents.RRN1138

**Published:** 2010-04-19

**Authors:** Jeffrey Shaman, Virginia Pitzer, Cecile Viboud, Marc Lipsitch, Bryan Grenfell

**Affiliations:** ^*^Oregon State University; ^†^Princeton University and NIH/FIC; ^‡^Fogarty International Center, National Institutes of Health, Bethesda, MD, USA and ^§^Princeton University

## Abstract

Much of the observed wintertime increase of mortality in temperate regions is attributed to seasonal influenza. A recent re-analysis of laboratory experiments indicates that absolute humidity strongly modulates the airborne survival and transmission of the influenza virus. Here we extend these findings to the human population level, showing that the onset of increased wintertime influenza-related mortality in the United States is associated with anomalously low absolute humidity levels during the prior weeks. We then use an epidemiological model, in which observed absolute humidity conditions temper influenza transmission rates, to successfully simulate the seasonal cycle of observed influenza-related mortality. The model results indicate that direct modulation of influenza transmissibility by absolute humidity alone is sufficient to produce this observed seasonality. These findings provide epidemiological support for the hypothesis that absolute humidity drives seasonal variations of influenza transmission in temperate regions.

 Note: The final version of this work appears in *PLoS Biology*.  

  **Introduction**

      In temperate regions, wintertime influenza epidemics are responsible for considerable morbidity and mortality[1].  These seasonal epidemics are maintained by the gradual antigenic drift of surface antigens, which enables the influenza virus to evade host immune response[2].  Recent influenza epidemics have resulted from the co-circulation of three virus (sub)types, A/H1N1, A/H3N2 and B, with one generally predominant locally in a given winter [3] [4] [5].  In contrast, influenza pandemic activity can occur any time of year, including during spring or summer months, in the rare instances when a novel virus to which humans have little or no immunity jumps from avian or mammalian hosts into the human population, as in the on-going H1N1v pandemic [6] [7] [8] [9]. Despite numerous reports describing wintertime transmission of epidemic influenza in temperate regions [10], our understanding of the mechanisms underlying influenza seasonal variation remains very limited.

      Experimental studies suggest that influenza virus survival within aerosolized droplets is strongly associated with the absolute humidity (AH) of the ambient air, such that virus survival improves markedly as AH levels decrease [11].  A similar relationship is observed between AH and airborne influenza virus transmission among laboratory guinea pigs, in that transmission increases markedly as AH levels decrease (Figure 1).  Within temperate regions of the world, AH conditions are minimal in winter and maximal in summer (Figure 1d).  This seasonal cycle favors a wintertime increase of both influenza virus survival and transmission, and may explain the observed seasonal peak of influenza morbidity and mortality during winter. Annual wintertime mortality peaks are evident in the long-term mortality records of excess pneumonia and influenza (P&I) in the US, a robust indicator of the timing and impact of epidemics at national and local scales [4] (Figure S1).   

**Figure 1.  ** Analyses of laboratory data, environmental data, and SIRS model simulations.  a) Linear and log-linear regression of guinea pig airborne influenza virus transmission data [12] [13] on specific humidity (a measure of AH);  b) Log-linear regression of 1-hour influenza virus survival data [14] on specific humidity; c) Functional relationship between \begin{equation*}R_{0} (t)\end{equation*} and \begin{equation*}q(t)\end{equation*} per Equation 4; d) 1972-2002 daily climatology of 2-meter above-ground NCEP-NCAR reanalysis specific humidity [15] for Arizona, Florida, Illinois, New York state and Washington state;  e) 1972-2002 average daily values of \begin{equation*}R_{0} (t)\end{equation*} derived from the specific humidity climatology using the best-fit parameter combination from SIRS simulations ( \begin{equation*}R_{0max}\end{equation*}= 3.52; \begin{equation*}R_{0max}\end{equation*}= 1.12) and the functional form (Figure 1c and Equation 4); f) Average\begin{equation*}R_{E} (t)\end{equation*} for all wintertime outbreaks in the 10 best-fit simulations at each state shown for 100 days prior through 150 days post outbreak onset (minimum 400 infections/day during 2 weeks prior; minimum 5000 infections/day at least one day during subsequent 30 days).  Figures 1a and b are redrawn from Shaman and Kohn[11]using specific humidity as the measure of AH. 

**Figure:**
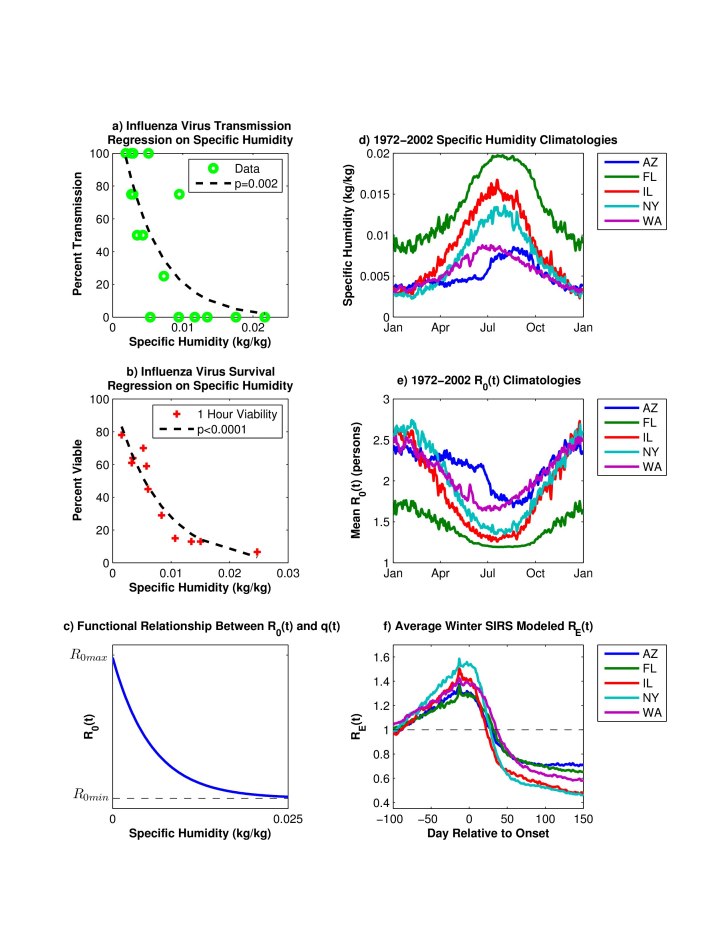

      Here we develop epidemiological support for these previous laboratory-based findings implicating AH as a driver of seasonal influenza transmission. First, we analyze the spatial and temporal variation of epidemic influenza onset across the continental US, 1972-2002, and correlate this observed variability with records of AH for the same period and locations.  Second, we show that a mathematical model of influenza transmission in the US can reproduce the spatial and temporal variation of epidemic influenza when daily AH conditions within each state are used to modulate the basic reproductive number,_\begin{equation*}R_{0} (t)\end{equation*}_, of the influenza virus.

**Results**

## AH and the Onset of Wintertime Influenza Outbreaks

      Our first test of the hypothesis that low AH drives wintertime increases of influenza transmission is to assess whether the onset of the influenza epidemic each winter – which shows substantial annual variation (Supplementary Figure S1; supplementary figures and text can be found here)—corresponds to a period of unusually low AH.  We define the onset of wintertime influenza as the date at which, for the 2 weeks prior, observed excess P&I mortality had been at or above a prescribed threshold level (e.g. 0.01 deaths/100,000 people).  This onset date was identified separately for each of the 30 winters in the 1972-2002 observational record at each of the 48 contiguous states plus the District of Columbia (DC).  We then examined the anomalous AH (\begin{equation*}AH'\end{equation*}) conditions prior to and following these onset dates. \begin{equation*}AH'\end{equation*}  is the local  daily deviation of AH from its 31-year mean for each day (as shown for 5 states in Figure 1d), defined as:

          **                                        **

\begin{equation*}AH' = AH - \bar{AH}\end{equation*}

     **(1)**

****

where \begin{equation*}\bar{AH}\end{equation*} denotes the 1972-2002 daily average value.  At temperate latitudes, such as in the US, wintertime AH levels are already much lower than summer (Figure 1d).   By using \begin{equation*}AH'\end{equation*} , we can determine whether the onset of wintertime influenza occurs when AH is above or below typical local daily AH levels.  

      Negative _\begin{equation*}AH'\end{equation*}  _ values are typically observed beginning 4 weeks prior to the onset of influenza epidemics (Figure 2), with the largest excursion occurring 17 days prior to onset. This result is robust to the choice of the mortality threshold level used to define onset date (from 0.001 to 0.02 excess P&I deaths/100,000 people). To assess the statistical significance of the association between negative wintertime \begin{equation*}AH'\end{equation*} and epidemic onset, we bootstrapped the distribution of observed wintertime \begin{equation*}AH'\end{equation*} records and found strong statistical support (*p*<0.0005, see Supporting Information, available here).  Depending on the threshold used to define onset, 55-60% of onset dates demonstrate negative \begin{equation*}AH'\end{equation*} averaged over the 4 weeks prior to onset.  While highly statistically significant, this shift from the expected 50% likelihood is small.  These findings indicate that negative \begin{equation*}AH'\end{equation*} are not necessary for wintertime influenza onset but instead presage an increased likelihood of these onset events.  In effect, negative \begin{equation*}AH'\end{equation*}  in the weeks prior to onset provide an additional increase of influenza virus survival and transmission over typical local wintertime levels and may further facilitate the spread of the virus.   

**Figure 2. **(top) Plots of \begin{equation*}AH'\end{equation*} averaged for the site-winters with an influenza outbreak showing the 6 weeks prior and 4 weeks following outbreak onset. The conditions at each of the site-winters are defined based on the onset date for that site-winter.  The onset dates are defined as the date at which wintertime observed excess P&I mortality had been at or above a prescribed threshold level for two continuous weeks  (e.g. 0.01 deaths/100,000 people).  Not every site-winter produced an outbreak as defined by a particular onset threshold.  Depending on the threshold level used, 1181-1420 epidemics were identified among 1470 possible (30 winters each for the 48 contiguous states plus DC).  Each solid line is the  averaged \begin{equation*}AH'\end{equation*} associated with influenza onset as defined by a different threshold mortality rate. The dashed line shows \begin{equation*}AH'\end{equation*} \begin{equation*}=0\end{equation*}. (bottom) Plot of \begin{equation*}R_{0} (t)\end{equation*} anomalies using the above \begin{equation*}AH'\end{equation*} values. The \begin{equation*}R_{0} (t)\end{equation*} anomalies are calculated using the best combined fit estimates of \begin{equation*}R_{0max}\end{equation*} and \begin{equation*}R_{0min}\end{equation*} (Table 1). The dashed line shows \begin{equation*}R_{0} (t)' = 0\end{equation*} . 

**Figure:**
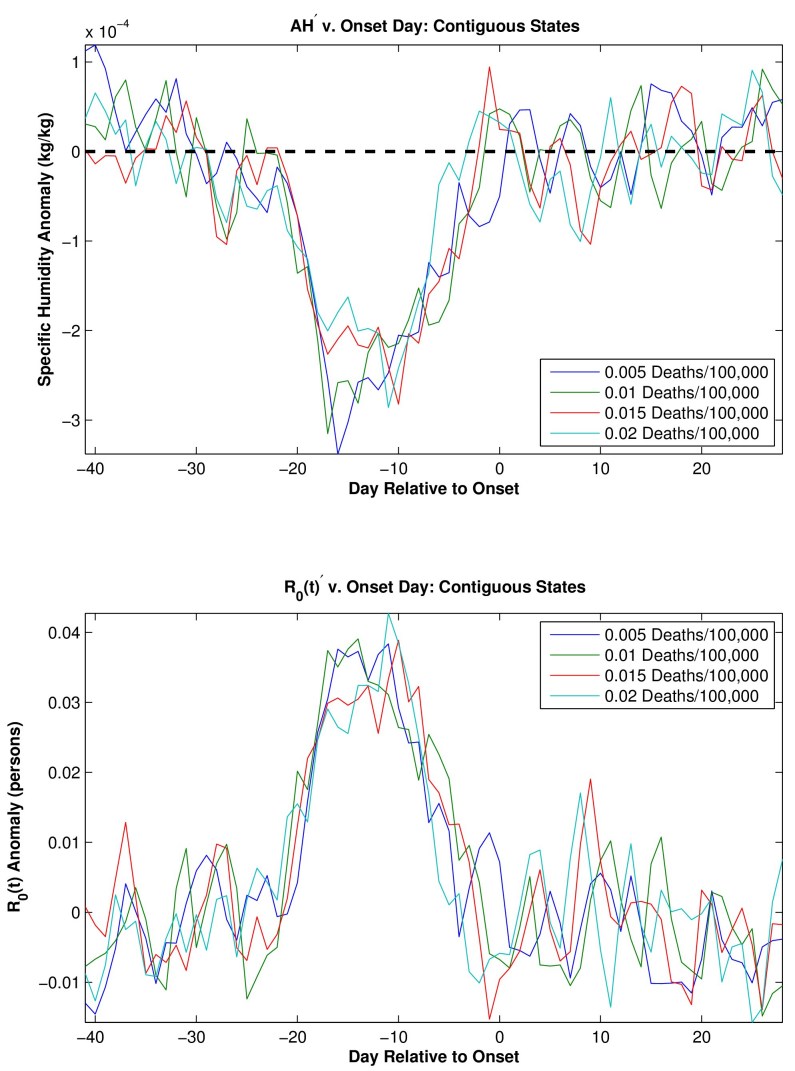

      Regional differences in the association of negative \begin{equation*}AH'\end{equation*} with onset date are also evident.  The association is strongest in the eastern US, in particular the Gulf region and the northeast (Figures S2-S3).  While the association does not reach statistically significance in much of the western US, \begin{equation*}AH'\end{equation*} are typically negative during the weeks prior to onset in this region as well.

      Next, we used the same approach to examine whether other potential environmental drivers of influenza are associated with wintertime influenza onset.   The findings indicate that negative relative humidity (RH) and temperature anomalies, as well as positive solar insolation anomalies, are also associated with onset date (Table 1). However, the direction of the associations of the daily wintertime anomalies of solar insolation and RH with epidemic onset are contrary to the association between these environmental factors and epidemic activity at the seasonal time scale.  Decreased solar insolation during the winter months is posited to increase influenza activity by decreasing host melatonin and vitamin D levels and thus host resistance[16] [17]; however, our findings indicate that influenza onset is associated with *increased* daily solar insolation anomalies. Similarly, RH is highest in winter [11], but influenza onset is associated with *low* RH anomalies.

**Table 1.  **Association of daily anomalies in various environmental variables with wintertime influenza onset during 1972-2002 for the contiguous US.   4 different onset thresholds are shown.  Average values for each variable are for the period 4 to 0 weeks prior to onset.  Significance estimates based on bootstrapping are also shown in parentheses.  NS = not significant.   

**Table:** 

**Onset Threshold** **(Deaths/100,000)**	**AH’ ** (1000*kg/kg)	**RH’** **(%)**	**Temperature’** **(Kelvin)**	**Solar Radiation’** **(W/m** ^**2**^ **)**
0.005	-0.138 (<0.00002)	-0.420 (0.00166)	-0.221 (0.00004)	0.431 (0.0397)
0.01	-0.124 (<0.00002)	-0.586 (0.00006)	-0.212 (0.00044)	0.547 (0.0068)
0.015	-0.114 (<0.00002)	-0.709 (<0.00002)	-0.178 (0.00398)	0.594 (0.0051)
0.02	-0.107 (<0.00002)	-0.639 (<0.00002)	-0.184 (0.00402)	0.316 (NS)

      Specific weather patterns may explain the observed correlations between these meteorological anomalies and influenza onset.  Anomalously low AH over the continental US is typically associated with excursions of colder air masses from the north.  These air masses, which often follow a cold front, bring cloud-free skies (i.e. increased solar insolation) and reduced surface temperature and humidity levels.  As the air mass moves southward, it slowly warms; however, unless it traverses a large open water source, AH does not increase substantially.  As a consequence, anomalously low RH levels can develop within these air masses as well.  Thus, the anomalies of solar insolation and RH could be non-causally linked with influenza outbreaks through their association with weather conditions that bring negative \begin{equation*}AH'\end{equation*} to a region.   

      Temperature and AH are strongly correlated (Table S1); both are minimal in winter when influenza transmission is maximal and have negative anomalies associated with influenza onset, tendencies which agree with the associations determined from laboratory data [11] [12] [13].   To establish which of these variables is most critical for onset, we rely on previous laboratory analyses exploring the impact of both environmental factors that indicate AH is the essential determinant of influenza virus survival and transmission [11].  Furthermore,__ \begin{equation*}AH'\end{equation*} is the only anomaly variable whose association with onset is significant at *p *< 0.00002 for all 4 onset threshold levels (Table 1).  

      In addition, it should be noted that seasonal temperature conditions are often highly managed indoors, where most of the U.S. population spends the bulk of its time.  Average daily outdoors temperatures can differ over 20°C from winter to summer, but seasonal heating and air conditioning greatly reduce this temperature cycle indoors. In contrast, AH possesses a large seasonal cycle both outdoors and indoors [11]. 

## Model Simulations of Influenza Seasonality

      To further assess the hypothesis that AH is a fundamental driver of influenza seasonality, we examined whether a population-level model of influenza transmission forced by AH conditions could reproduce the observed seasonal patterns of P&I mortality. We simulated influenza transmission for 5 states representative of different climates within the US: Arizona, Florida, Illinois, New York and Washington. The model considers 3 disease classes: susceptible, infected, recovered; to integrate the impact of waning immunity following antigenic drift, we allow individuals to go back to the susceptible class at a defined rate (SIRS model). Observed 1972-2002 daily AH conditions within each state are used to modulate the basic reproductive number,\begin{equation*}R_{0} (t)\end{equation*}, of the influenza virus, i.e. the per generation transmission rate in a fully susceptible population. These daily fluctuations of \begin{equation*}R_{0} (t)\end{equation*} alter the transmission probability per contact within the SIRS model and thus affect influenza transmission dynamics.  The SIRS model contains 4 free parameters: 2 (_\begin{equation*}R_{0max}\end{equation*}_ and \begin{equation*}R_{0min}\end{equation*}) that define the range of \begin{equation*}R_{0} (t)\end{equation*}, 1 for the duration of immunity (*D*), and 1 for the duration of infectiousness (*L*).

      If absolute humidity controls influenza seasonality, best-fit simulations with the AH-driven transmission model should meet the following criteria: 1) the mean annual model cycle of infection should match observations in each state; 2) these simulations should converge to similar parameter values, i.e. the virus response to AH should be consistent among states; and 3) AH modulation of transmission rates ( \begin{equation*}R_{0} (t)\end{equation*}) within the model must match the large range implied by the laboratory data (Figure 1). 

      Multiple 31-year (1972-2002) simulations were run at each of the 5 states with randomly chosen parameter combinations. We then compared the mean annual cycle of daily infection from each simulation with a similar average of 1972-2002 observed excess P&I mortality rates [3] [4].  Best-fit model simulations at each site capture the observed seasonal cycle of influenza (Figure 3).  These simulations not only produce the late-year rise in transmission and infection, but also the wintertime peak during early January, typically followed by a secondary peak during late February/early March.  In both models and observations, the dual winter peaks are not typically seen in individual years; rather these epidemic trajectories reflect the averaging of individual wintertime outbreaks that peak anytime between December and April (Figure S5).

**Figure 3.  ** Mean annual cycles for the best-fit SIRS model simulations at the 5 state sites.  Here, best-fit simulations were selected individually for each state based on RMS error after scaling the 31-year mean daily infection number to the 31-year mean observed daily excess P&I mortality rate.  Thick blue line shows the best-fit simulation; thinner green lines show the next 9 best simulations. 

**Figure:**
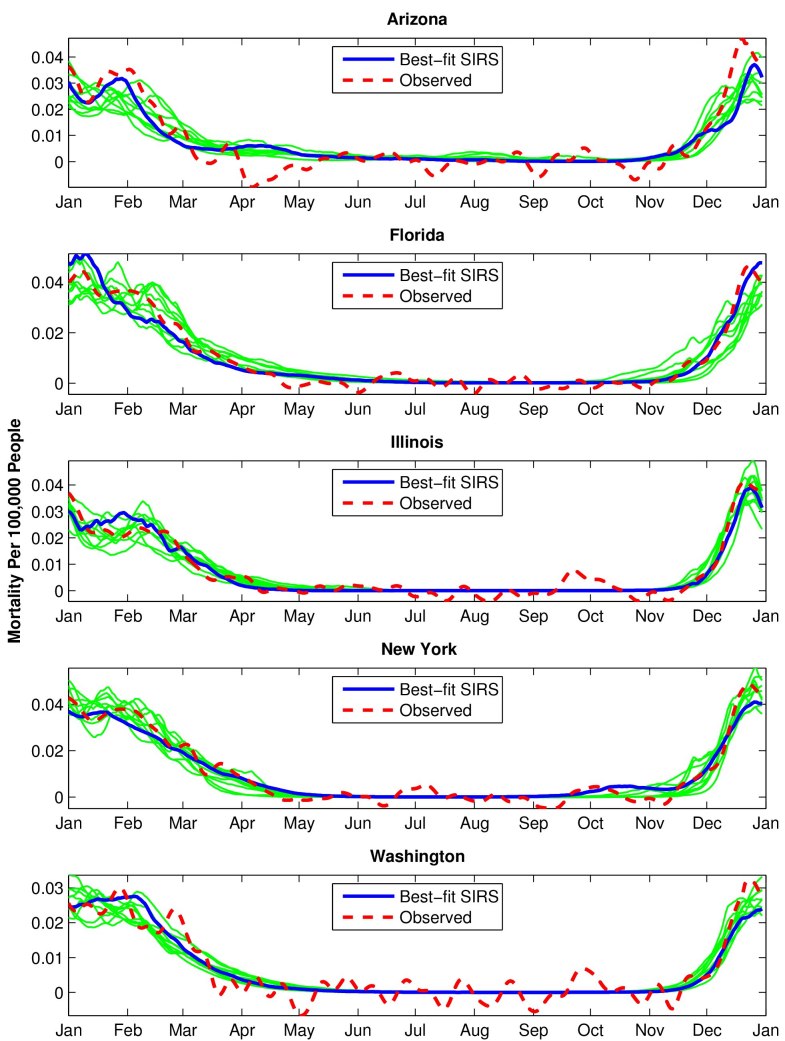

      We also searched for the best-fit parameter combinations for all 5 sites evaluated together.  The parameter combinations of these best ‘combined fits’ are characterized by high \begin{equation*}R_{0max}\end{equation*} (generally >2.8), high \begin{equation*}R_{0min}\end{equation*} (>1) and low mean infectious period (2<*D*<4.2 days) (Table 2, Figure S6).  Best-fit simulations at each of the 5 sites individually occupy a similar parameter space (Table S2, Figures S7-S11).  In particular, these simulations converge to high \begin{equation*}R_{0max}\end{equation*} , which indicates a similar response to AH variability (see Supporting Information).    

**Table 2.  **Parameter combinations for the 10 best-fit simulations at the Arizona, Florida, Illinois New York, and Washington state sites.  5000 simulations were performed at each site with the parameters \begin{equation*}R_{0max}\end{equation*}, \begin{equation*}R_{0min}\end{equation*}, *D * and *L* randomly chosen from within specified ranges.  Best-fit simulations were selected for the 5 sites in aggregate based on RMS error after scaling the 31-year mean daily infection number to the 31-year mean observed daily excess P&I mortality rate at each site.  The scaling factor itself, representing mortality per infection is also shown.    

**Table:** 

**Rank**	**RMS Error**	**Correlation Coefficient** **(r)**	**L (years)**	**D (days)**	\begin{equation*}R_{0max}\end{equation*} (persons/person)	\begin{equation*}R_{0min}\end{equation*} (persons/person)	**Scaling Factor** **(x1e-4)**
1	0.0070	0.85	5.35	3.24	3.52	1.12	1.70
2	0.0070	0.85	5.40	2.41	2.89	1.16	1.92
3	0.0075	0.83	3.28	4.18	3.40	1.22	1.04
4	0.0075	0.82	3.70	2.03	2.05	1.15	1.85
5	0.0075	0.82	7.77	2.59	3.69	1.30	2.28
6	0.0076	0.82	6.23	2.37	2.71	1.23	2.28
7	0.0076	0.82	6.05	2.56	3.79	1.06	1.83
8	0.0076	0.82	4.61	2.71	2.61	1.29	1.70
9	0.0076	0.81	7.39	2.85	3.69	1.27	2.22
10	0.0076	0.81	3.58	3.61	3.19	1.20	1.18

      There is some correlation among SIRS model parameter values in simulations that fit the observed excess P&I mortality well.  For instance, among better-fit simulations, *L * and *D *tend to be inversely related (Figures S6-S11).  In addition, broad regions of parameter space appear capable of producing high-quality, low-RMS-error simulations (Figure S6).  The stochastic components of the SIRS model may contribute in part to this behavior.  The flat goodness-of-fit within model parameter space indicates that no one parameter combination is strictly ‘best’; rather, a range of parameter combinations may produce good simulations of influenza transmission.  These parameter ranges are: *L* = 3-8 years, *D* = 2-3.75 days, \begin{equation*}R_{0max}\end{equation*} = 2.6-4, and \begin{equation*}R_{0min}\end{equation*} = 1.05-1.30.  We re-ran the SIRS model repeatedly sampling this approximate subset range of parameter space.  Best-fit simulations from this subset range of parameter space (Table S3) were of similar quality and exhibited the same flat goodness-of-fit within model parameter space as the best-fit simulations presented in Table 2.

      Because the SIRS model simulates only influenza-related infections, not deaths, a scaling factor is needed to compare model-simulated rates of infection with the observed excess P&I mortality rates.  This scaling factor can be understood as the case fatality ratio, i.e. the probability of mortality given infection.  Reassuringly, all best-fit simulations produce a scaling factor of the same order of magnitude and roughly consistent with the expected value of the case fatality ratio for P&I-related deaths (see Supporting Information).  

      The model also explains regional variations in influenza dynamics.  Due to the modeled nonlinear relationship between \begin{equation*}R_{0} (t)\end{equation*} and AH (Figure 1c), the seasonal cycle of \begin{equation*}R_{0} (t)\end{equation*}  is sensitive to both AH seasonal cycle amplitude and mean AH levels (Figures 1d-e).   In Florida, mean AH levels are higher than for the other 4 states, but the seasonal AH cycle remains large and produces a seasonal \begin{equation*}R_{0} (t)\end{equation*} cycle of sufficient amplitude to generate an effective reproductive number, \begin{equation*}R_{E} (t)\end{equation*} = \begin{equation*}R_{0} (t)\end{equation*} \begin{equation*}*S(t)/N\end{equation*} , greater than 1 (Figure 1f) and organize influenza epidemics preferentially during winter.  Outbreak dynamics reinforce this phase organization in that wintertime epidemics confer immunity to a large proportion of the model population, which then reduces population-level susceptibility during the following summer when \begin{equation*}R_{0} (t)\end{equation*}  is low.  In Arizona and Washington state, the seasonal AH cycle is less than for the other 3 states, but average AH levels are low, where laboratory findings indicate sensitivity to variation in AH is greater; consequently \begin{equation*}R_{0} (t)\end{equation*} retains a sizeable seasonal cycle (Figure 1e).  For all 5 states the AH-driven seasonal variation of \begin{equation*}R_{0} (t)\end{equation*} is large enough that \begin{equation*}R_{E} (t)\end{equation*} is strongly modulated by AH conditions and exceeds 1 during winter as outbreaks develop (Figure 1f).  

      The humidity-driven SIRS simulations satisfy our 3 criteria for supporting the hypothesis that AH controls influenza seasonality in temperate regions.  The simulations produce a consistent response in the 5 climatologically diverse US states using similar parameter values.  The large sensitivity of simulated influenza transmission to AH is consistent with the analysis of laboratory experiments that show large changes in influenza virus survival and transmission in response to AH variability (Figures 1a,b).  

*Cross Validation of the Model Findings*

      To further validate the SIRS model findings, we determined whether the best-fit simulations derived from the 5 selected states could reproduce the seasonal cycles of influenza elsewhere in the US.  The 10 best combined-fit parameter combinations (Table 2) were used to perform 31-year (1972-2002) SIRS simulations at each of the contiguous 48 states plus DC.  

      The results of this cross-validation demonstrate good simulations of observed excess P&I mortality for a majority of states (average *r* > 0.7, minimum *r *> 0.5, see Methods and Table 3).  Some states, particularly the sparsely populated western states perform less well.  These states often have low workflow [4], which may reduce the rate of introduction of the virus each winter. In addition, heterogeneous AH fields across some states (particularly large ones) create some error due to simulation with a single average statewide AH value. 13 states in the continental US, including Arizona, possess low workflow rates [4].  6 of these 13 states are among the 10 worst cross-validation performers; such a clustering is unlikely to occur by chance alone (*p* < 0.005).   In addition, 7 of the 10 worst performers are states with the 10 lowest population densities (*p *< 0.0001).    

**Table 3.** Correlation coefficients for the contiguous US and District of Columbia of SIRS-simulated 1972-2002 influenza incidence with 1972-2002 observed excess P&I mortality.  The 10 best common-fit parameter combinations (Table 1) were used for these hindcast projections. Results are ordered based on best average correlation (among the 10 simulations for each state).  States marked with an asterisk are low workflow states.  The 10 states with lowest 1972-2002 population density are shown in bold.

**Table:** 

	**10-Run**	**Table 1 Ranked Parameter Combination**									
**State**	**Average**	**1**	**2**	**3**	**4**	**5**	**6**	**7**	**8**	**9**	**10**
MO	0.92	0.93	0.95	0.87	0.89	0.91	0.93	0.85	0.93	0.95	0.97
OK*	0.91	0.94	0.95	0.05	0.96	0.84	0.90	0.88	0.85	0.91	0.93
KS	0.90	0.96	0.89	0.84	0.85	0.89	0.92	0.89	0.92	0.91	0.91
KY	0.89	0.91	0.88	0.87	0.90	0.83	0.88	0.88	0.95	0.87	0.92
AR	0.88	0.88	0.88	0.86	0.90	0.91	0.89	0.89	0.84	0.93	0.86
IA	0.88	0.91	0.88	0.92	0.81	0.91	0.91	0.86	0.85	0.89	0.87
PA	0.88	0.88	0.91	0.85	0.82	0.90	0.96	0.93	0.89	0.80	0.87
NH*	0.88	0.90	0.85	0.88	0.85	0.80	0.95	0.91	0.85	0.89	0.93
NY	0.88	0.88	0.82	0.92	0.78	0.87	0.85	0.94	0.95	0.96	0.81
IL	0.87	0.95	0.92	0.87	0.95	0.73	0.80	0.95	0.94	0.76	0.79
MA	0.86	0.89	0.86	0.87	0.93	0.69	0.77	0.90	0.87	0.90	0.97
IN	0.86	0.95	0.90	0.78	0.95	0.84	0.80	0.82	0.81	0.92	0.86
VA	0.86	0.80	0.75	0.83	0.81	0.93	0.84	0.93	0.79	0.94	0.94
NC	0.85	0.75	0.86	0.85	0.90	0.85	0.81	0.88	0.95	0.74	0.96
AL	0.85	0.83	0.84	0.85	0.97	0.69	0.90	0.89	0.97	0.76	0.85
MI	0.85	0.74	0.90	0.90	0.76	0.89	0.89	0.72	0.89	0.92	0.89
WV	0.84	0.83	0.84	0.90	0.86	0.92	0.81	0.84	0.79	0.86	0.75
WI	0.84	0.86	0.73	0.86	0.85	0.70	0.89	0.78	0.89	0.92	0.91
**NE**	0.84	0.91	0.78	0.78	0.87	0.94	0.87	0.67	0.81	0.93	0.81
ME*	0.84	0.72	0.91	0.76	0.86	0.86	0.92	0.79	0.81	0.89	0.86
TN	0.84	0.88	0.83	0.88	0.78	0.72	0.81	0.85	0.92	0.86	0.83
AZ*	0.82	0.69	0.80	0.85	0.82	0.82	0.90	0.84	0.86	0.81	0.84
CO*	0.82	0.85	0.90	0.80	0.84	0.60	0.86	0.72	0.86	0.92	0.91
MS	0.82	0.76	0.82	0.78	0.60	0.97	0.78	0.83	0.92	0.88	0.89
OH	0.82	0.76	0.87	0.91	0.79	0.79	0.76	0.66	0.80	0.91	0.91
TX	0.81	0.66	0.78	0.87	0.92	0.78	0.75	0.87	0.81	0.86	0.79
SD*	0.81	0.87	0.84	0.69	0.83	0.87	0.83	0.66	0.79	0.84	0.84
MN	0.81	0.84	0.72	0.74	0.81	0.83	0.85	0.80	0.76	0.86	0.84
MD	0.80	0.74	0.80	0.84	0.85	0.79	0.81	0.84	0.76	0.81	0.80
SC	0.80	0.80	0.84	0.89	0.84	0.76	0.92	0.55	0.84	0.86	0.74
WA	0.78	0.91	0.81	0.60	0.86	0.85	0.84	0.74	0.67	0.79	0.73
VT	0.77	0.68	0.84	0.87	0.76	0.76	0.87	0.72	0.73	0.64	0.82
GA	0.76	0.69	0.80	0.81	0.80	0.86	0.87	0.68	0.59	0.76	0.79
CT	0.76	0.75	0.75	0.83	0.76	0.71	0.66	0.87	0.80	0.65	0.83
LA	0.74	0.45	0.72	0.75	0.58	0.82	0.89	0.76	0.85	0.79	0.73
NM*	0.73	0.77	0.89	0.53	0.73	0.71	0.77	0.77	0.82	0.64	0.71
FL	0.71	0.64	0.75	0.74	0.73	0.57	0.73	0.78	0.71	0.70	0.77
DC	0.69	0.72	0.73	0.69	0.72	0.65	0.71	0.74	0.68	0.71	0.59
CA	0.68	0.74	0.57	0.63	0.61	0.69	0.78	0.52	0.67	0.79	0.77
UT*	0.68	0.68	0.74	0.66	0.59	0.68	0.77	0.75	0.63	0.55	0.73
NJ	0.67	0.74	0.67	0.82	0.56	0.69	0.48	0.71	0.60	0.62	0.85
RI	0.64	0.78	0.68	0.73	0.75	0.51	0.49	0.69	0.34	0.67	0.76
**OR**	0.62	0.79	0.58	0.59	0.73	0.63	0.44	0.68	0.79	0.56	0.43
ID*	0.62	0.65	0.74	0.62	0.63	0.75	0.61	0.30	0.63	0.69	0.53
WY*	0.59	0.69	0.57	0.55	0.59	0.71	0.67	0.43	0.65	0.43	0.64
DE	0.56	0.44	0.66	0.55	0.47	0.65	0.65	0.41	0.52	0.52	0.68
MT*	0.55	0.54	0.55	0.37	0.75	0.50	0.63	0.59	0.61	0.47	0.52
ND*	0.55	0.61	0.67	0.53	0.62	0.37	0.60	0.47	0.64	0.42	0.57
NV*	0.40	0.33	0.46	0.26	0.42	0.54	0.67	0.17	0.19	0.50	0.47

      Overall, the cross validation shows that the best combined-fit parameter combinations can simulate influenza seasonality throughout the country. Future use of higher resolution AH and observed P&I data that better represent local conditions may improve these model results.

*Additional SIRS Model Results*

      We also used SIRS model simulations to provide additional support for the association between negative \begin{equation*}AH'\end{equation*} and epidemic onset (Figure 2).  Best-fit SIRS model runs reveal a comparable effect in which large negative \begin{equation*}AH'\end{equation*} develop about 2 weeks prior to onset as defined by SIRS model infection rates (Figure 4). The 1-week difference in lag between this analysis with model infection rates (2 weeks) and the analysis with observed excess P&I mortality rates (3 weeks) roughly corresponds to the mediantime from infection to mortality[18] [19] [20].  The broader peak of negative \begin{equation*}AH'\end{equation*} seen in Figure 2 is likely due to other, real-world factors that affect onset response and are not represented in the SIRS model (see Supporting Information).

****

**Figure 4.** Plots of average \begin{equation*}AH'\end{equation*} associated with wintertime influenza onset for 10 best-fit SIRS model simulations at the 5 state sites (Arizona, Florida, Illinois, New York and Washington).  The onset dates are defined as the date on which wintertime infection rates have been at or above a prescribed level for two continuous weeks  (e.g. 50 infections/100,000 people).  Each solid line is the averaged \begin{equation*}AH'\end{equation*} associated with influenza onset as defined by a different threshold infection rate. The dashed line shows \begin{equation*}AH'\end{equation*} \begin{equation*}=0\end{equation*}. bottom) Plots of \begin{equation*}R_{0} (t)\end{equation*} anomalies using the \begin{equation*}AH'\end{equation*} values.  The \begin{equation*}R_{0} (t)\end{equation*}  anomalies are calculated using the parameters \begin{equation*}R_{0max}\end{equation*} and \begin{equation*}R_{0min}\end{equation*}  from each best-fit simulation (Table S3). The dashed line shows \begin{equation*}R_{0} (t)' = 0\end{equation*}. 

**Figure:**
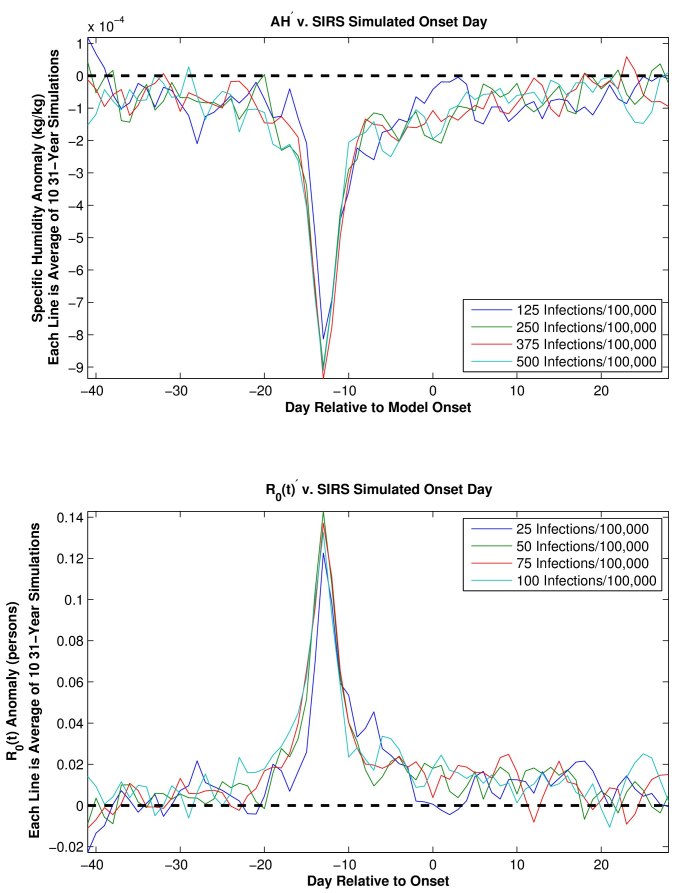

      Finally, we examined whether the school calendar, which alters person-to-person contact rates, could provide a better simulation of seasonal influenza than AH.  School holidays have been estimated to lead to changes of ~25% in influenza transmission [21]and occur during summer in the US, as well as at the end of the calendar year and again in spring.  A number of SIRS model simulations were performed that included a step-wise increase of \begin{equation*}R_{0} (t)\end{equation*} during the school year (see Supporting Information). Simulations in which school closure was the only modulation of \begin{equation*}R_{0} (t)\end{equation*}  were able to reproduce the seasonal cycle of influenza; however, these simulations did not reproduce observed excess P&I mortality as well as those with AH alone (see Supporting Information, Table S5, Figures S14-S15).  In addition, a 40-90% change in influenza transmission (\begin{equation*}R_{0} (t)\end{equation*}) was needed to effect this seasonality (Table S5).  This range of \begin{equation*}R_{0} (t)\end{equation*} changes is slightly larger than the previously estimated modulation of ~25%; however, these previous estimates were derived from an age-structured population model, so direct comparison is difficult.  

## **Discussion**

      Distinguishing among potential environmental drivers of influenza seasonality, such as AH, RH, temperature, solar insolation, and the school calendar, is difficult since all demonstrate a similarly strong annual periodicity.  Nevertheless, our findings indicate that AH is a major (and likely the predominant) determinant of influenza seasonality due to: 1) the empirical association of negative \begin{equation*}AH'\end{equation*} with the onset of wintertime influenza outbreaks (Figure 2), which is statistically stronger than for RH, temperature or solar insolation (Table 1); 2) the relative consistency of the response to AH among the 5 states modeled in detail (i.e. similar parameter space; Supplementary Table S2); and 3) the SIRS cross validation showing that the same best-fit parameters (Table 2) can produce successful simulations of influenza seasonality throughout much of the US (Table 3).

      In addition, several findings undermine the hypothesis that the association between the seasonal influenza cycle and AH is in fact due to confounding by other potential drivers.  The case for solar insolation is weakened by its implausible positive association with wintertime influenza onset.  While laboratory analyses find that low RH favors influenza virus survival and transmission, RH is in fact typically incorrectly phased in the outdoor environment (i.e. maximal during winter, minimal during summer) and cannot explain peak wintertime influenza incidence.  The case for temperature is weakened by the small amplitude of its seasonal cycle in most indoor environments. Finally, re-analyses of laboratory experiments indicate that AH is the best single-variable constraint of influenza virus survival and transmission[11]; associations with temperature and RH likely merely reflect their positive covariability with AH at various time scales.  Still, a role for temperature or other (possibly multiple) covariable factors cannot be entirely discounted.  Further laboratory investigation is needed to determine the effects of humidity, evaporation and temperature on virus protein structure and survival.

      SIRS model simulations also indicate that while the school calendar can explain seasonal epidemic influenza, the correspondence with observations is not as good as for simulations driven by AH (Figures S14-S15).  The required increase in transmissibility during school terms is greater than estimated previously; with such large variation in transmission, inclusion of non-summer breaks creates a noticeable decline in transmission in the Christmas and spring periods that is not observed in data (see Supporting Information). Nonetheless, an effect of school closure on influenza transmission rates is well-documented [21] [22] and cannot be discounted.  It is certainly possible that the effects of AH and the school calendar on influenza transmission act in concert with one another; however, our statistical and SIRS model findings indicate that AH variability provides a more parsimonious explanation for the seasonality of epidemic influenza in temperate regions, and, in addition, is associated with the onset date of individual wintertime outbreaks. The argument that AH at least partly determines influenza seasonality is supported by: 1) laboratory evidence [11]; 2) the much weaker seasonality in the tropics where humidity is high year-round, but a school calendar exists; 3) the \begin{equation*}AH'\end{equation*}-onset analysis (Figures 2 and 4); 4) the plausibility of parameter combinations and the effect size for AH within SIRS model simulations (Tables 2; Figures 1 and 3); and 5) the superior overall quality of AH-forced simulations (Figure S14) and their reduced sensitivity to stochastic processes within the SIRS model (Figure S15).

      There are minor differences among the sites in the best-fit parameter values for the SIRS model (Table S2 and Figures S7-S11), some of which could be host mediated.  For instance, Florida and New York show a tendency toward lower duration of immunity.  This difference could be derived from a number of host-mediated factors specific to these states.  The findings presented here do not preclude an influence of such factors on influenza transmission and seasonality*.*  Differences in population susceptibility and infectivity (e.g. population age and general health), seasonal variations of host behavior (e.g. more time indoors in close contact during winter [21]), and host resistance (e.g. wintertime melatonin or vitamin D deficiencies [16] [17]) may still affect influenza transmission rates*.*

      Among states there are also differences of average peak SIRS-simulated \begin{equation*}R_{E} (t)\end{equation*} (Figure 1f); however, there is no systematic relationship between rates of observed excess P&I deaths and those peak \begin{equation*}R_{E} (t)\end{equation*}  values among these sites.  For instance, Florida and New York have similar rates of observed excess mortality per 100,000 persons, but different peak \begin{equation*}R_{E} (t)\end{equation*} levels.  State-to-state differences in contact rates and population age and structure, in particular the proportion of seniors, who are at highest risk of influenza-related death during seasonal epidemics, undoubtedly affect influenza infection and mortality rates and modulate the amplitude and duration of individual outbreaks.  In addition, the influenza dominant subtype is a key predictor of influenza-related mortality rate each season; A/H3N2-dominant seasons are associated with 2-3 times higher death rates than H1N1 and B-dominant [3] [4]. These other factors are not accounted for in our SIRS model; hence, there is not a good, one-to-one correspondence between the average peak size of \begin{equation*}R_{E} (t)\end{equation*} and rates of observed excess P&I deaths.  However, within the SIRS model a relationship between \begin{equation*}R_{E} (t)\end{equation*} and simulated infection rates does exist.*  * Among the 10 best-fit simulations at each site, the average maximum \begin{equation*}R_{E} (t)\end{equation*} rank (from greatest to least) as: NY, IL, WA, AZ, FL.  Similarly among these runs, the average maximum epidemic size ranks (from greatest to least) as: NY, WA, IL, AZ, FL.  This more direct response is not unexpected as within the SIRS model, higher \begin{equation*}R_{E} (t)\end{equation*} directly corresponds to greater transmission and, consequently, more rapidly developing, larger outbreaks.

      It should be noted that observed excess P&I mortality is an imperfect indicator of influenza incidence, as other respiratory illnesses exhibit similar seasonal periodicities.  No doubt these other diseases contribute to the seasonality of the observational time series used here (Figure 3, Figure S1).  However, excess P&I mortality generally shows a strong correspondence with other indicators of influenza incidence, such as hospitalization data and laboratory notifications [4].  A clearer picture of the environmental determinants of influenza seasonality and onset will emerge when the effects of AH and other environmental variables on these potentially confounding, seasonal respiratory pathogens are also elucidated.  

      The initial evidence demonstrating that AH affects influenza virus survival and transmission was derived from laboratory experiments studying the airborne transmission of influenza; however, our SIRS model uses no specific mode of transmission.  Thus other modes of transmission, in particular indirect transmission via fomites, if similarly affected by AH, might also have a role determining the seasonality of influenza in temperate regions.  In addition, the SIRS model is highly idealized and fails to represent many factors in the real world that can affect transmission rates, including clustered populations, structured interactions, variation in host infectiousness, and multiple influenza strains conferring various levels of cross-immunity.  Future work could incorporate these effects into a more realistic influenza model that also accounts for the effects of AH.  Such efforts would also enable better discrimination between the effects of school terms and AH. Also, the effects of AH on influenza transmission should be incorporated into models accounting for travel and workflow [4] [23] [24] to explain the seasonal geographic spread of influenza.  

      The analyses presented here need to be extended elsewhere, including the tropics where AH is high year-round and the seasonality of influenza is often less clearly defined.  High AH does not preclude but merely reduces influenza virus survival and transmission, so it is possible a role for AH also exists in the tropics.  However, the findings presented here suggest that \begin{equation*}R_{0} (t)\end{equation*} would be less sensitive to AH variability in areas of very high year-round AH, such as the tropics, which may allow for other, possibly host-mediated, factors to play a more predominant role in generating seasonal variability in influenza incidence.

      Laboratory studies provided the initial evidence that AH may determine the seasonality of influenza in temperate regions [11].  The model and statistical results presented here indicate that the effects of AH observed in the laboratory are sufficient to explain patterns observed at the population level and illustrate the power of epidemiological modeling rooted in individual-level experiments.  The results indicate that AH affects both the seasonality of influenza incidence and the timing of individual wintertime influenza outbreaks in temperate regions.  The association of negative with wintertime influenza outbreak onset is remarkable given the noise in the data and suggests that skillful, short-term probabilistic forecasts of epidemic influenza could be developed.

**Materials and Methods**

## SIRS Model and Methods

      The SIRS model equations are:

\begin{equation*}\frac{dS}{dt} =\frac{N-S-I}{L} -\frac{\beta(t)IS }{N}\end{equation*}

         **(2)**

_\begin{equation*}\frac{dl}{dt} =\frac{\beta (t)IS}{N} -\frac{I}{D}\end{equation*}_ **(3)**

where *S * is the number of susceptible people in the population, *t * is time in years, *N *is the population size, *I * is the number of infectious people, *N*-*S*-*I * is the number of resistant individuals, _\begin{equation*}\beta (t)\end{equation*}_ is the contact rate at time *t*, *L * is the average duration of immunity in years, and *D * is the mean infectious period in years. The basic reproductive number at time *t *is \begin{equation*}R_{0} (t)\end{equation*} \begin{equation*}=\beta (t) D\end{equation*}.

      Observed AH conditions were derived from NCEP-NCAR reanalysis [15].  For each state (Arizona, Florida, Illinois, New York and Washington), a daily 1972-2002 time series of 2-m above-ground specific humidity, \begin{equation*}q(t)\end{equation*} , was constructed by averaging all grid cells with ≥10% of their area within that state.  The equation relating  \begin{equation*}q(t)\end{equation*} to \begin{equation*}R_{0} (t)\end{equation*} uses a exponential functional form similar to the relationships between AH and both influenza virus survival and transmission, derived from laboratory experiments (Figure 1):

                                          \begin{equation*}R_{0} (t)= exp (a \times q(t)+ b) +R_{0min}\end{equation*} (4)

****

where , *a *= -180, \begin{equation*}b= log( R_{0max} -R_{0min} )\end{equation*},    \begin{equation*}R_{0max}\end{equation*} is the maximum daily basic reproductive number, and \begin{equation*}R_{0min}\end{equation*} is the minimum daily basic reproductive number.  The value of *a *is estimated from the laboratory regression of influenza virus survival upon AH (Figure 1a).  Equation 4 dictates that 

\begin{equation*}R_{0} (t)\end{equation*} \begin{equation*}=\end{equation*} \begin{equation*}R_{0max}\end{equation*} when \begin{equation*}q(t) = 0 \frac{kg}{kg}\end{equation*} and that \begin{equation*}R_{0} (t)\end{equation*} approaches \begin{equation*}R_{0min}\end{equation*}asymptotically as \begin{equation*}q(t)\end{equation*} increases.

      For simulations, we use a stochastic Markov chain formulation in which individuals are treated as discrete entities, and transitions between model states (i.e. susceptible, infected, recovered) are determined by random draws corresponding to rates determined from Equations 2 and 3.   Using the daily time series of \begin{equation*}q(t)\end{equation*}, which alters \begin{equation*}R_{0} (t)\end{equation*}, daily influenza transmission was simulated for each of the 5 state sites during 1972-2002 for a model population of 500,000 individuals.  Initial conditions included 50,000 susceptible persons and 100 infected persons; however, results were not sensitive to these numbers.  Simulations were performed with daily random seeding of infected individuals (i.e. each day there is a 10% probability that a single susceptible individual becomes infected), meant to represent re-introduction of the virus in the model domain due to travel.  The model is perfectly mixed and simulations were performed with two influenza virus subtypes: A-H1N1/B and A-H3N2.   No cross immunity was conferred within the model between these virus subtypes.  Each year beginning in May, the random seeding of infectious individuals in the population (representing emigration/travel) is fixed to the dominant recorded subtype for the US (i.e. either A-H1N1/B or A-H3N2), based on CDC/MMWR laboratory and antigenic surveillance data [3] [4].

      5000 simulations at each site were run using combinations of the 4 model parameters: \begin{equation*}R_{0max}\end{equation*},\begin{equation*}R_{0min}\end{equation*}, *D *and *L *chosen using a Latin hypercube sampling structure with uniform distribution.  The ranges of these parameters were specified to reflect known influenza dynamics.  Estimates of \begin{equation*}R_{0}\end{equation*} derived or used by many authors ranges from 1.3 to 3 [18] [23] [24] [25] [26] [27] [14].  To effect this range, given Equation 3 and variations of \begin{equation*}q(t)\end{equation*}, we used an \begin{equation*}R_{0max}\end{equation*} ranging from 1.3 to 4. \begin{equation*}R_{0min}\end{equation*} provides the \begin{equation*}R_{0}\end{equation*} below which the model cannot fall. This minimum recognizes that other modes of influenza transmission exist that may not modulated by absolute humidity. \begin{equation*}R_{0min}\end{equation*} values range from 0.8 to 1.3.  Per Equation 4, decreasing humidity increases \begin{equation*}R_{0} (t)\end{equation*}.  The range of this non-linear increase is set by the randomly-chosen \begin{equation*}R_{0max}\end{equation*} and \begin{equation*}R_{0min}\end{equation*} parameters.  Estimates of *D * range from 2 to 7 days [18] [28], and estimates of *L * range from 2 to 10 years.  Both influenza virus subtypes used the same 4 randomly-chosen parameters during each simulation, though results were similar when each subtype was assigned different parameters (8 in total).  

      The quality of each simulation at each site was evaluated based on RMS error with observed excess pneumonia and influenza (P&I) mortality [3] [4] (Figure S1**)**, lagged 2 weeks.  The lag accounts for mean time from infection to mortality.  Prior to determining the RMS error each model run was scaled to enable comparison of simulated infections with observed mortality rates (see Supporting Information).

      Prior to the model cross-validation throughout the contiguous U.S., we first determined the effect that stochasticity within each SIRS simulation has on the quality of fit with observations.  We re-ran the 10 best common-fit parameter combinations 100 times each for New York, each time with different random seeding, and found that correlations to observed P&I mortality ranged from *r * = 0.65 to *r *= 0.97 with an average of *r * = 0.87.  In contrast, multiple simulations with the 4500^th^ best parameter combination, out of 5000, produced correlations that ranged from *r *= -0.18 to *r * = 0.34. Thus, random seeding within a particular model run produces a range of correlation coefficient outcomes; however, good model runs should produce high positive correlation with observations (average * r* > 0.7, minimum *r *> 0.5).

*NOTE: Supporting Information including tables and figures (whose numbers include the letter S) is available by clicking here. Warning: the file is large and will take some time to download over slow connections.*

**Funding**

      This work was supported in part by the US National Institutes of Health Models of Infectious Disease Agent Study program through cooperative agreements 5U01GM076497 (M. L.) and 1U54GM088588 (M. L. and J. S.).  V. E. P. and B. G. were supported by NIH grant R01GM083983-01, the Bill and Melinda Gates Foundation, the RAPIDD program of the Science and Technology Directorate, US Department of Homeland Security, and the Fogarty International Center, NIH.  The funders had no role in study design, data collection and analysis, decision to publish, or preparation of the manuscript.   

**Competing Interests**

 M.L. discloses consulting income from the Avian/Pandemic Flu Registry (Outcome Sciences, funded in part by Roche) and from Novartis Vaccines and Diagnostics. The other authors declare no competing interests.  
